# Does adoption of superior Murrah buffalo germplasm pay off? evidence from a causal impact study in Haryana, India

**DOI:** 10.3389/fgene.2025.1713072

**Published:** 2026-01-12

**Authors:** Makarabbi Gururaj, S. Aiswarya, Chhotaray Supriya, Sanjay Kumar

**Affiliations:** 1 Transfer of Technology (ToT) Unit, ICAR-CIRB, Hisar, Haryana, India; 2 Animal Genetics and Breeding (AGB) Division, ICAR-CIRB, Hisar, Haryana, India

**Keywords:** field progeny testing, impact, inverse probability weighted regression adjustment, Propensity Score Matching and Coarsened Exact Matching Murrah, network project on Buffalo improvement, superior germplasm

## Abstract

Buffaloes play a pivotal role in sustaining rural livelihoods, milk production, and nutritional security in South Asia, where genetic improvement remains a critical pathway to enhance productivity and profitability. Despite decades of breeding efforts, limited empirical evidence exists on the socio-economic impact of disseminating superior germplasm under structured progeny testing programs. This study employed a quasi-experimental design to evaluate the effects of the Central Institute for Research on Buffaloes (CIRB) Murrah superior germplasm disseminated under the Field Progeny Testing (FPT) program of the Network Project on Buffalo Improvement (NPBI) in Hisar district, Haryana, India. Treatment households were defined as adopters of CIRB superior germplasm, while non-adopters from comparable control villages, located approximately 40–50 km away to avoid spill-over, served as the control group. A total of 200 respondents (100 treatment and 100 control) were selected through a combination of purposive and random sampling. Data were collected using key informant interviews, focus group discussions, and structured household surveys, and analysed using doubly robust econometric approaches, including Inverse Probability Weighted Regression Adjustment (IPWRA), Propensity Score Matching (PSM), and Coarsened Exact Matching (CEM). Our findings suggest that calf birth weight, age at first calving, dry period, average daily milk yield, and cost of artificial insemination significantly influence the net income of buffalo-rearing households. Adoption of CIRB Murrah superior germplasm through the FPT program led to a significant higher annual net income by $405.94 per buffalo, average daily milk yield by 1.53 kg, and calf birth weight by 5.53 kg, while reducing Age at First Calving (AFC) by 2.94 months and shortening dry periods by 1.83 months in comparison with control group. The study further indicates that efficient reproductive management and affordable AI services are critical for realizing the full economic potential of buffalo herds. Consequently, the findings emphasize that targeted dissemination of superior germplasm combined with technical guidance on feeding, breeding, and health management is essential for enhancing productivity and profitability in dairy systems.

## 1 Introduction

Livestock production is a foundation of global agriculture, sustaining the livelihoods of over 1.3 billion people and contributing to food and nutritional security, rural employment, and poverty alleviation. In India, livestock plays a more critical role, providing a regular cash flow, supporting mixed farming systems, and serving as a safety net against agrarian risks. Within the livestock sector, dairy animals play a central role in household nutrition and income security, particularly for small-scale farmers. India is home to 109.85 million buffaloes, which represent 20.45 per cent of the national livestock population and contribute about 103.30 million tonnes of milk annually (DAHD, 2024). The genetic diversity of the buffalo population reflects both descript and non-descript types, with descript breeds accounting for 56.63 per cent (Kumar et al., 2019). Among these, the Murrah buffalo stands out, representing 44.39 per cent of the descript animals, followed by Surti (3.58%), Mehsana (3.33%), Jaffrabadi (1.68%), and Bhadawari (1.61%) (DAHD, 2019). Murrah buffaloes, native to Haryana, are globally recognised for their high milk yield, reproductive efficiency, and adaptability (Kumar et al., 2019). They are used as an improver breed within India and internationally, where their germplasm has been exported to enhance local buffalo populations. The centrality of Murrah buffaloes to both India’s dairy economy and global buffalo improvement programs underscores the urgent need for systematic breeding interventions. The availability of genetically superior and performance-tested bulls remains a significant constraint in buffalo breeding. This scarcity hampers the systematic improvement of non-descript populations and slows genetic progress compared to cattle. Superior germplasm, particularly from progeny-tested bulls, is crucial for enhancing productivity, reducing the age at first calving, improving disease resistance, and ensuring the long-term sustainability of buffalo-based dairy systems. A coordinated breeding programme that combines institutional resources with farmer participation is critical for achieving these goals.

### 1.1 Network project on buffalo improvement (NPBI)

Network Project on Buffalo Improvement, launched in 1993, aims to produce, evaluate, and disseminate genetically superior bulls across India (Network Project on Buffalo Improvement, 2023). ICAR–Central Institute for Research on Buffaloes, Hisar, serves as the coordinating centre. Initially covering five Murrah centres, the project later expanded to include Jaffrabadi, Surti, Bhadawari, and Nili-Ravi breeds, as listed in Table 1. NPBI operates through a network of ICAR institutes, agricultural universities, and field units, integrating elite herd management, breed conservation, and field-level progeny testing (Central Institute for Research on Buffaloes, 2024).

**TABLE 1:** Participating centres under NPBI.

S. No.	Name of centre	Breed	Year of start
ICAR institute			
I	ICAR-CIRB, Hisar	Murrah	1993
II	ICAR-NDRI, Karnal	Murrah	1993
III	ICAR-IVRI, Izatnagar	Murrah	1993
IV	ICAR research Complex, Eastern region, Patna	Murrah	2014
V	ICAR-CIRB, Sub-Campus, Nabha	Nili-Ravi	2001
VI	ICAR- IGFRI, Jhansi	Bhadawari	2001
Animal Science/Agricultural University			
I	LUVAS, Hisar	Murrah	1993
II	GADVASU, Ludhiana	Murrah	1993
III	GADVASU, Ludhiana	Nili Ravi	2018
IV	KU, Junagarh	Jaffarabadi	2001
V	RAJUVAS, LRS, Vallabhnagar	Surti	2001
Field unit			
I	ICAR-CIRB, Hisar	Murrah	2001
II	ICAR-NDRI, Karnal	Murrah	2001
III	GADVASU, Ludhiana	Murrah	2001

NDRI: National Dairy Research Institute, IVRI: Indian Veterinary Research Institute, IGFRI: Indian Grassland and Fodder Research Institute, LUVAS: Lala Lajpath Rai University of Veterinary and Animal Sciences, GADVASU: Guru Angad Dev Veterinary and Animal Science University, KU: Kamdhenu University, RAJUVAS: Rajasthan University of Veterinary and Animal Sciences

The primary objectives of NPBI are to:

1. To establish the elite nucleus herd of important breeds for the producing genetically superior quality bulls.
2. Evaluation of sire/superior bulls through institutional/associated herd/field progeny testing program.
3. Produce, test, propagate and conserve high genetic merit male germplasm.
4. Create institutional-farmer linkages for large-scale dissemination of improved germplasm.

### 1.2 Field progeny testing (FPT) programme under NPBI

The Field Progeny Testing Programme, initiated in 2001–02, was a landmark intervention of NPBI aimed at evaluating bull performance through the productivity of their daughters under farmers’ conditions (NPBI annual report 2022-23). At CIRB Hisar, about 4,000 artificial inseminations (AIs) are carried out annually with semen from superior Murrah bulls. Over the past 2 decades, 72,717 AIs have been performed, yielding 35,430 pregnancies with an average conception rate of 51.10%. Out of these, 23,532 calvings were recorded, including 11,372 female progenies that formed the base for bull evaluation. A significant constraint has been the premature sale of 11,898 pregnant buffaloes before calving, which reduced the effective number of progenies available for evaluation (NPBI annual report 2022-23). Nevertheless, the programme has delivered substantial benefits to farmers, such as higher milk productivity, reduced age at first calving, and access to superior progeny-tested bulls. It has also facilitated the development of elite herds and generated significant genetic gains at the field level.

While the biological achievements of NPBI and the FPT programme are well-documented, the economic dimensions remain un-quantified (Ullah and Waheed, 2025; Rege et al., 2011; Van Eenennaam et al., 2014). Despite clear evidence of farmer-level benefits in productivity and reproductive efficiency, no systematic study has measured the monetary benefits accrued to farmers and society. This gap is significant because large-scale genetic improvement programmes require substantial public investment, and their sustainability depends on demonstrable socio-economic returns. In contrast, cattle breeding programmes in several countries have been subjected to rigorous economic evaluations (Kariuki et al., 2017; Okeno et al., 2010; König et al., 2009) which provided evidence for policymaking and resource allocation. However, such economic studies are virtually absent in the context of buffaloes, especially for Murrah progeny testing under Indian conditions. The lack of evidence makes it difficult to prioritise investments and design policies that maximise both genetic gain and farmer welfare.

Given this background, the present study is pioneering in its attempt to quantify the economic impact of the dissemination of superior quality Murrah germplasm through FPT Programme under NPBI on the incomes of buffalo farmers. This study provides critical insights into the effectiveness of public investments in buffalo breeding by systematically assessing the benefits realised by farmers. It also creates a methodological foundation for evaluating similar genetic improvement programmes in other livestock species. Hence, the specific objective of this study is to assess the economic impact of the adoption of superior quality Murrah germplasm in field conditions on the income of buffalo farmers.

## 2 Materials and methods

### 2.1 Research design

The study employed a quasi-experimental research design to assess the impact of the dissemination of CIRB Murrah superior germplasm on farmers’ income and buffalo productivity. Farmers who adopted the CIRB Murrah superior germplasm through the FPT program under the NPBI were classified as the treatment group, while farmers using other germplasm for artificial insemination (AI) were designated as the control group. Control villages were selected based on multiple factors, including socio-economic status, agro-climatic conditions, breed type, veterinary infrastructure, asset holdings, and geographical distance (approximately 40–50 km) from the treatment villages, ensuring comparability and minimizing potential spill-over effects. The study follows a cross-sectional quasi-experimental framework and relies on selection on observables to identify programme impacts.

### 2.2 Study area and sample description

The study was conducted in Hisar district of Haryana, India, under the FPT program of the NPBI, coordinated by ICAR–CIRB, Hisar. Haryana was selected due to its strategic importance in Murrah buffalo rearing, high prevalence of dairy buffalo households, and well-established veterinary and artificial insemination infrastructure, making it a representative setting to evaluate the impact of superior germplasm dissemination. Hisar district was specifically chosen as it hosts CIRB, the coordinating institute for the FPT program, and encompasses a large number of villages actively engaged in buffalo improvement initiatives, facilitating both treatment and control group identification.

### 2.3 Sampling plan

**FIGURE 1:**
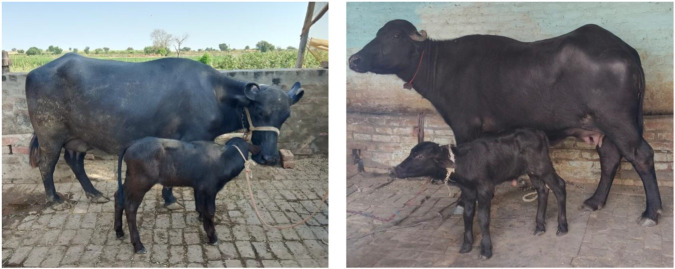
Calf born through CIRB superior Murrah germplasm.

The study employed a combination of purposive and random sampling techniques to select respondents. Initially, villages participating in the FPT program under the NPBI were purposively chosen as treatment villages, where CIRB Murrah superior germplasm had been disseminated (Figure 1). The selected treatment villages included Bichpari, Jewra, Juglan, Bado Patti, Khedi Barkhi, Bir Babran, Sarsod, Dhiktana, Bugana, Kirara, and Shymsukh. Control groups were randomly selected from nearby villages with comparable socio-economic status, agro-climatic conditions, breed type, veterinary infrastructure, and asset holdings, but which had not adopted CIRB superior germplasm. The selected control villages included Muklan, Dabra, Bhodha Dhani, Thaska, Jandawala Bagar, Chaharwala, Dabri, Ramsara, Kishanpuriya, and Janana, located approximately 40–50 km from the treatment villages to minimize potential spill-over effects. In the second stage, ten farmers were randomly selected from each village in both treatment and control groups, resulting in a total of 200 respondents (100 from treatment villages and 100 from control villages).

### 2.4 Data collection

The study employed multiple methods to collect comprehensive and reliable data, including Key Informant Interviews (KII), Focused Group Discussions (FGDs), and individual household surveys. This multi-method approach enabled triangulation of information and ensured both qualitative insights and quantifiable data for robust statistical analysis.

#### 2.4.1 Key informant interviews (KII)

KIIs were conducted prior to the FGDs and household surveys. Key informants were selected based on their active involvement in buffalo production and related activities. These included veterinary surgeons, Veterinary and Livestock Development Assistants (VLDAs) from the State Animal Husbandry Department, village sarpanches, private veterinary practitioners, and progressive farmers. The KIIs provided detailed information on village-level characteristics, including livelihood options, land utilization, cropping patterns, livestock inventory, and veterinary infrastructure and services. Furthermore, insights from KIIs helped refine and validate parameters related to productive and reproductive traits, which were later incorporated into the structured household survey.

#### 2.4.2 Focused group discussions (FGDs)

A total of four FGDs were organized, two each in treatment and control villages. Both male and female farmers were participated, with group sizes ranging from 12 to 21 participants. Each FGD was facilitated by the local Veterinary and Livestock Development Assistant. The discussions focused on obtaining detailed information about livelihood options in village, land utilization, cropping patterns, livestock inventory, status of cattle and buffalo husbandry practices, productive and reproductive performance, and veterinary services. FGDs also served to validate and refine the parameters for productive and reproductive traits, ensuring that the household survey captured relevant and locally contextualized data.

#### 2.4.3 Household survey

Following the KIIs and FGDs, a structured household survey was conducted to collect standardized and quantifiable information from dairy farmers. The survey aimed to facilitate statistical comparison between treatment and control groups and to generate evidence on the impact of CIRB Murrah superior germplasm on buffalo productivity and household income. The interview schedule was designed with three distinct parts: face sheet information, cost and returns information, and impact component.

The first section, the face sheet, gathered background information on the socio-economic and farm characteristics of each respondent. It covered demographic features such as age, gender, education, family size, and family composition, along with dairy farming experience and primary occupation. Farm-level details such as total landholding, area under fodder cultivation, and type of buffalo housing were also recorded. Information on livestock inventory was also compiled, detailing the number of buffaloes and cows by category (in-milk, dry, heifer, male and female calves) and ownership of CIRB progenies. These variables provided essential context for understanding differences in adoption behaviour and herd structure between the treatment and control groups.

The second section, focusing on cost and returns from buffalo rearing, aimed to document the economic performance of the enterprises. A budgetary analysis framework was applied to estimate total and net income from buffalo milk production. Data were collected on costs incurred for feed and fodder (including concentrates, dry and green fodder, and mineral mixture), labour (both hired and family), veterinary and breeding services such as AI, vaccination, and treatment, as well as miscellaneous expenses like repairs, water, electricity, and housing. The output side captured milk yield per day, total lactation yield, and the prevailing milk sale price. Income from dung, sale of male calves, and culling of adult buffaloes was also recorded.

The final section focused on assessing the impact of CIRB Murrah germplasm on productivity, reproduction, and profitability indicators. This component analyzed the differential performance between the treatment and control groups using variables such as birth weight of calf, age at first calving, dry period, service period, calving interval, number of AIs per conception, average cost per AI, average milk yield per day, sale price of adult buffalo, and annual net income per animal.

### 2.5 Analytical technique

#### 2.5.1 Cost and returns from buffalo rearing

The net income from buffalo milk production was estimated using budgetary analysis. Total costs included expenditures on feed and fodder, labor (hired and family), veterinary services (AI, deworming, treatment, vaccination), and miscellaneous items such as repairs, water, and electricity. Buffalo units were converted into Standard Animal Units (SAU) following Sirohi et al. (2019) for cost standardization. Feed and fodder costs were calculated using purchase prices or, for homegrown inputs, their imputed values. Labor costs were computed based on time spent on buffalo management multiplied by prevailing wage rates. Farm income included sales of milk and milk products, dung, male calves (6–12 months), and adult buffaloes. Milk income was calculated as total lactation yield multiplied by the sale price per kg. Dung income was estimated from total quantity of dung produced per herd using SAU conversion and sale price in village, with imputed values for dung used on the farm. Male calves were valued at market price, while female calves retained for milk production were assigned an imputed value based on prevailing prices.

#### 2.5.2 Impact evaluation

The impact of adopting CIRB Murrah superior germplasm on buffalo productivity and farmer income was quantified using the Average Treatment Effect on the Treated (ATT), defined as:

$$ATT=E\left[Y_{i}^{A}-Y_{i}^{\left\{NA\right\}}|T_{i}=1\right]$$

Where:

- E denotes the expectation operator,
- Y_i_^A^ represents the potential outcome for the *i*th adopter household (T*i* = 1), and
- (Y*i* ^NA^) represents the potential outcome for the (*i*)-th non-adopter household (T*i* = 0).

A critical challenge in estimating ATT is that the counterfactual outcome of adopters had they not adopted $\left[Y_{i}^{A}-Y_{i}^{\left\{NA\right\}}|T_{i}=1\right]$ cannot be directly observed. Substituting outcomes of non-adopters for this counterfactual may result in biased ATT estimates (Takahashi and Barrett, 2014).

#### 2.5.3 Inverse probability weighted regression adjustment (IPWRA)

To address potential selection bias, the study employed the IPWRA method, a doubly robust estimator that combines propensity score weighting with regression adjustment (Wooldridge, 2010). This method provides consistent ATT estimates even if either the treatment or outcome model is mis-specified. The outcome model estimates the effect of treatment on net income, while weighting adjusts for observable differences between groups.

The outcome model was specified as:

$$Y_{i}=\alpha +\beta T_{i}+\gamma X_{i}+\epsilon_{i}$$

Where:

- Y_i_ = net income per buffalo per annum,
- T_i_ = treatment indicator (1 for adopters, 0 for non-adopters),
- X_i_ = vector of covariates (birth weight, age at first calving, milk yield, dry period, service period, AI per conception),
 - $\epsilon_{i}$ = stochastic error term.

#### 2.5.4 Propensity score estimation

Propensity scores were estimated using pre-treatment household and farm characteristics to ensure covariate balance between treatment and control groups.

$$P\left(T_{i}=1|X_{i}\right)=\Phi \left(X_{i}\theta \right)$$

Where Φ is the cumulative distribution function of the standard normal distribution, and θ is a vector of parameters.

#### 2.5.5 ATT estimation

The ATT was computed as:

$$ATT=1/\left\{n_{A}\right\}\sum_{\left\{i\in A\right\}}\left(\;Y_{i}-{r\hat{\;}}^{\left\{NA\right\}}\left(X_{i}\right)\right)$$

Where:

- (n_A_) = number of adopters,
-
${r\hat{\;}}^{\left\{NA\right\}}\left(X_{i}\right)$ = predicted outcome for non-adopters based on observed covariates, Similarly, regression coefficients for adopters (δ^A^= (α^A^, β^A^); non-adopters (δ^NA^= (α^NA^, β^NA^) were estimated using inverse probability-weighted least squares.

#### 2.5.6 Robustness and sensitivity analysis

To ensure robustness, additional estimation methods were applied (Choudhary et al., 2024):

1. Propensity Score Matching (PSM): matches treated and control households based on similar propensity scores to reduce selection bias and achieve covariate balance.
2. Coarsened Exact Matching (CEM): matches treated and control households by coarsened covariates to reduce imbalance and model dependence.

## 3 Results

### 3.1 Socio-economic profile of the respondents

The household serves as the primary beneficiary of buffalo rearing technology, making household characteristics such as age, education status, family size, and farming experience crucial considerations in the adoption process. Additionally, farm characteristics such as land holding, fodder production, along with institutional and market factors, play a significant role in influencing adoption. The disparities in socio-economic characteristics between households from treatment and control groups are evident from Table 2. Specifically, respondents in the treatment group were, on average, older (46.64 years) than those in the control group (43.27 years). Similarly, the treatment group had higher experience in dairy farming (24.29 years) compared to the control group (21.45 years). Literacy levels were slightly higher in the treatment group (79%) than in the control group (77%), indicating relatively better access to education among adopters. Household size was marginally larger in the treatment group (4.89 members) than in the control group (4.69 members). Interestingly, average land holdings were smaller in the treatment group (3.16 acres) compared to the control group (6.50 acres), whereas land under fodder production was slightly higher among adopters (0.56 acres vs. 0.47 acres), suggesting a focus on forage cultivation despite smaller holdings. Furthermore, households in the treatment group owned an average of 2.36 CIRB progenies, which were absent in the control group. The average sale price of milch buffaloes was higher for the treatment group ($1146.21) than the control group ($937.02), reflecting differences in herd quality and market returns.

**TABLE 2:** Socio-economic profile of the respondents.

Parameters		Treatment group		Control group	
		Mean	Std. error	Mean	Std. error
Age of the respondent (Years)		46.64 (21–82)	1.25	43.27 (22–70)	1.1
Experience in dairy farming (Years)		24.29 (03–06)	0.95	21.45 (05–50)	0.88
Educational level	Literate	79%	-	77%	-
	Illiterate	21%	-	23%	-
Family size (In No.)		4.89 (02–14)	0.22	4.69 (02–12)	0.19
Average land holdings (acres)		3.16 (0–18)	0.41	6.50 (0–60)	1.35
Average land under fodder production (acres)		0.56 (0.18–1.02)	0.05	0.47	0.04
Average no. of CIRB progenies (milch buffalo) owned by the respondents (In No.)		2.36 (01–06)	0.17	-	-
Average sale price of the milch buffaloes (In Rupees)		$1146.21 ($551.06 - $2534.89)	3,950	$937.02 ($440.85 - $2479.79)	3,480

Figures in parenthesis indicates the range value for the respective parameter.

### 3.2 Livestock inventory of the respondents

Livestock resources form a critical component of farm productivity and income, especially in buffalo-rearing households. The inventory of animals among the treatment and control groups provides insights into herd composition and potential production capacity, which are essential considerations in evaluating the effects of improved buffalo management practices. Table 3 presents the average number of buffaloes and cows per household across different categories such as in-milk, dry, heifer, and calves. On average, households in the treatment group possessed slightly fewer in-milk buffaloes (1.09) than those in the control group (1.14), while the number of dry buffaloes was lower in the treatment group (0.27) compared to the control group (0.42). The number of heifers was marginally higher in the treatment group (0.83 vs. 0.78). Male and female calves were slightly fewer in the treatment group, indicating a slightly smaller young stock compared to non-adopters.

**TABLE 3:** Livestock inventory (average number per household).

S. No.	Type of animal	Treatment group		Control group	
		Buffaloes	Cows	Buffaloes	Cows
1	In-milk	1.09	0.09	1.14	0.42
2	Dry	0.27	0.06	0.42	0.19
3	Heifer	0.83	0.12	0.78	0.14
4	Male calf	0.44	0.04	0.61	0.07
5	Female calf	0.64	0.02	0.81	0.26
Total		**3.27**	**0.33**	**3.76**	**1.08**

The bold represents the average values of the treatment and control group.

### 3.3 Choice of variables and its descriptive statistics

The selected parameters covering productive, reproductive, and economic traits and their mean value differences for the control and treatment groups are presented in Table 4. The analysis revealed statistically significant differences in variables such as birth weight of calves, age at first calving (AFC), dry period, average cost per artificial insemination (AI), daily average milk yield, average sale price of the adult buffalo and average annual net income per buffalo. Other reproductive parameters, including service period, calving interval, and AI per conception, were statistically non-significant.

**TABLE 4:** Choice of variables and their mean value difference among control and treatment groups.

Variables	Control	Treatment	Difference
Birth weight of calf (In kgs)	34.29	39.82	−5.53***
Age at first calving (AFC) of the buffalo (In months)	44.42	41.48	2.94**
Dry period of the buffalo (In months)	4.99	3.16	1.83**
Average cost per AI	$4.01	0.050	$3.96***
Average milk yield of buffalo per day (In kg)	6.92	8.45	−1.53*
Average sale price of the adult buffalo (in-milk and dry)	$1146.21	$937.02	$209.19***
Service period (In months)	4.73	4.47	0.26
Calving interval (In months)	15.02	14.42	0.60
AI per conception (In No.)	2.82	2.63	0.19
Annual net income per buffalo	$368.03	$773.97	-$405.94***

*, ** and *** indicate significance at 0.1, 0.05 and 0.01 level respectively.

Calves born through the use of CIRB superior quality Murrah semen exhibited a significantly higher birth weight in the treatment group (39.82 kg) compared to the control group (34.29 kg; p < 0.01). The higher birth weight can be attributed to better management and balanced feeding of advanced pregnant animals, which directly influences calf health and subsequent reproductive performance. The age at first calving was significantly reduced in the treatment group (41.48 months) compared to the control group (44.42 months), reflecting enhanced reproductive efficiency (p < 0.05). Furthermore, the dry period was shorter among the treated buffaloes (3.16 months vs. 4.99 months in the control group), indicating improved lactation length management and greater potential for annual milk yield. The treatment group demonstrated superior performance in milk productivity. The average daily milk yield was 8.45 kg per buffalo, significantly higher than 6.92 kg in the control group (p < 0.05). The improvement in both productive and reproductive parameters resulted premium market price for adult buffaloes of treatment group ($1146.21) as compared to control group (₹ 85,019). The premium market price and enhanced productive and reproductive parameters in the treatment groups are attributable to the use of genetically superior Murrah semen under the CIRB Field Progeny Testing programme and the accompanying technical guidance on feeding, breeding, and health management. The cost of artificial insemination was considerably lower for the treatment group ($0.050) compared to the control group ($4.01; p < 0.01), highlighting the cost-effectiveness of the FPT programme. In terms of net returns, the annual net income per buffalo was substantially higher in the treatment group ($773.97) compared to the control group ($368.03), with a difference of $405.94 per buffalo per annum (p < 0.01).

### 3.4 Factors influencing net income of the respondents

**FIGURE 2:**
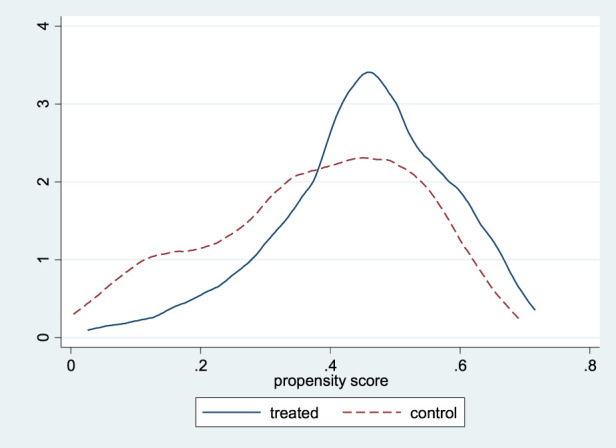
Common support for identification of comparable Beneficiaries-FPT (treatment) and Non-Beneficiaries-Non-FPT (control group).

To examine how productive, reproductive, and economic traits are associated with household net income from buffalo rearing, a probit regression model was estimated using the matched sample. Importantly, this model was not used for causal identification or treatment assignment, but as an exploratory analysis to understand income-relevant performance attributes. The variables included in this model such as birth weight, age at first calving, milk yield, service period, and cost per AI are outcome-related indicators influenced by management and genetic improvement. Therefore, the results are interpreted as associative rather than causal.A visual inspection of the common support region (Figure 2) clearly indicates a substantial overlap in the distribution of propensity scores across the two groups, thereby confirming that the assumption of common support holds firmly.

The findings presented in Table 5 indicate that several productive and reproductive parameters significantly influenced the net income of participating buffalo-rearing households. Birth weight of calves had a positive and significant effect, with adoption probabilities increasing by 0.144, implying that higher calf birth weights contribute to improved income outcomes. Conversely, age at first calving (AFC) exhibited a negative effect (−0.068), reflecting the economic penalty associated with delayed reproductive maturity. Similarly, a longer service period (−0.140) and higher cost per AI (−0.118) were negatively associated with household income, highlighting the importance of efficient reproductive management and affordable breeding services. On the other hand, average daily milk yield demonstrated a strong and positive association (0.157) with net income, reaffirming that productivity enhancement is the most direct pathway to profitability. The effect of calving interval was positive though modest (0.075), suggesting that households maintaining optimal intervals benefit from better income realization. Other variables, such as AI per conception, dry period, and average sale price of adult buffalo, were statistically insignificant but showed expected directional effects.

**TABLE 5:** Probit model estimates of factors influencing net income under Field Progeny Testing program.

Variable	Coefficient (B)	Std. error	Marginal effect
Birth weight (BW)	3188.79**	7586.32	0.144
Age at first calving (AFC)	−1604.81*	4326.67	−0.068
Dry period (DDP)	−7165.66	16391.59	−0.048
Cost per AI (CAMP)	−6412.64*	5717.69	−0.118
Average milk yield per day (AMY)	14614.02**	10415.50	0.157
Average sale price of adult buffalo (PVB)	−0.036	0.074	−0.051
Service period (SP)	−15829.23**	12678.47	−0.140
Calving interval (CI)	7489.01*	11468.01	0.075
AI per conception (AI)	−25893.50	28535.97	−0.100
Log likelihood	−165.42		
LR χ^2^ (12)	48.33*		
Matched sample size	188 (96 control, 92 treated)		

### 3.5 Estimation of causal impact

The results of Table 6 indicate that the adoption of CIRB superior quality Murrah buffalo semen through the FPT program had a significant positive impact on the annual net income per buffalo. Specifically, the ATT results from the IPWRA model reveal that households in the treatment group earned, on average, $405.94 more per buffalo per annum compared to the control group. The ATE estimates across the different approaches (IPWRA, PSM, and CEM) consistently show a significant increase in net income ranging from $390.47 to $394.97, confirming the robustness of the impact across matching and weighting techniques. Causal inference is derived exclusively from these ATT estimates under the assumption of selection on observables and correct temporal ordering of treatment and outcomes. These results suggest that the field progeny testing program effectively enhanced the economic returns of buffalo-rearing households. The observed increase in net income can be primarily attributed to improvements in productive and reproductive performance of the buffaloes, including higher milk yield, shorter dry periods, better birth weight of calves, and age at first calving as reflected in the earlier analysis (Table 5). The consistency between ATT and ATE estimates across multiple methods further reinforces that the treatment effect is systematic and not an artefact of model specification.

**TABLE 6:** Treatment Effect on outcome variable (annual net income).

Method	ATE estimate ($)	Robust SE	ATT estimate ($)	Robust SE
IPWRA	$391.27**	7.29	$405.94**	7.42
PSM	$394.97**	8.21	$404.89**	8.45
CEM	$390.47**	8.55	$402.54**	8.02

** and *** indicate significance at 0.05 and 0.01 level respectively.

ATE: Average Treatment Effect; ATT: Average Treatment effect on the Treated; CEM: Coarsened Exact Matching; IPWRA: Inverse Probability Weighted Regression Adjustment; PSM: Propensity Score Matching.

## 4 Discussion

The findings of this study provide robust evidence on the transformative impact of field progeny testing under Network Project on Buffalo Improvement in strengthening buffalo-based dairy systems. The adoption of superior quality Murrah semen, coupled with technical handholding, generated a chain of improvements spanning reproductive efficiency, productivity enhancement, and economic viability. Importantly, these gains were not merely confined to biological parameters but extended to household-level economic outcomes, reflecting the interlinkages between technology adoption, resource optimization, and farm-level decision-making (McDonald et al., 2016; Kaushik and Rajwanshi, 2025).

The socio-economic characteristics of participating households highlight the relevance of demographic and institutional factors in shaping adoption dynamics. Households in the treatment group tended to be more experienced in dairying and demonstrated marginally higher literacy levels, which suggests that knowledge access and accumulated farming wisdom act as enablers of technology adoption. This pattern aligns with earlier observations that education, experience, and family resources are pivotal in the uptake of genetic improvement technologies, as they lower the perceived risk and enhance absorptive capacity (Luh et al., 2014; Micheels and Nolan, 2016; Akinsulu et al., 2025). Interestingly, adopters possessed smaller landholdings but allocated proportionally more area for fodder cultivation, indicating a deliberate strategy to align feed resources with improved animal requirements. This echoes the argument by Choudhary et al., 2024 that progressive dairy households often prioritize forage production over land expansion, reflecting an intensification pathway where efficiency gains outweigh scale advantages.

Differences in livestock inventory between treatment and control groups, while modest in absolute numbers, hold interpretive value. Adopters maintained slightly fewer lactating animals (cattle and buffaloes) but compensated with higher productive efficiency per animal, suggesting that FPT interventions shifted the production frontier from herd expansion to yield intensification. This observation resonates with findings by Adesogan et al., 2025, who reported that genetic and managerial interventions often reduce the need for larger herds, as improvements in per-animal yield reduce pressure on household resources. The observed composition, with fewer young stocks among adopters, may reflect a conscious culling strategy driven by confidence in genetic merit, where households retain fewer but superior-quality progenies (Woods, 2017). Such herd structuring indicates a qualitative transformation in buffalo husbandry practices, moving away from subsistence-oriented maintenance to market-responsive management.

The reproductive and productive performance parameters offer compelling evidence of the biological efficacy of the FPT program. Calves born through CIRB superior semen exhibited higher birth weights, which is not only a proxy for genetic gain but also indicative of improved maternal nutrition and perinatal management (Jenkins et al., 2016; Mwangi et al., 2025). Early reproductive maturity, reflected in reduced age at first calving, and shorter dry periods collectively enhance lifetime productivity, lowering the inter-calving interval and increasing the number of productive lactations (Eulmi, 2024). These shifts are consistent with reports that structured progeny testing accelerates genetic progress and improves reproductive indices in dairy herds (Brito et al., 2021). The increase in daily milk yields among treated households directly reinforces the role of FPT in addressing India’s persistent challenge of low buffalo productivity, a constraint long identified as limiting dairy sector growth despite the species’ dominance in national milk output (Kona et al., 2025). Importantly, these gains were achieved under field conditions, stressing the external validity of the program beyond controlled farm trials.

Economic outcomes strongly mirrored biological improvements, validating the economic rationale for public investment in FPT. Higher market prices for buffaloes from treatment households point to the premium attached to genetically superior and more productive animals. Net annual income per buffalo among adopters was more than double that of non-adopters, emphasizing the profitability of the program. Such outcomes resonate with the conclusions of De Vries (2017) who argued that genetic interventions, when coupled with improved management practices, yield disproportionate economic returns relative to costs. Particularly notable was the dramatic reduction in the cost of artificial insemination in the treatment group, highlighting how institutional delivery mechanisms under FPT reduced transactional barriers and democratized access to advanced genetics. This reduction not only alleviated financial constraints but also expanded the reach of breeding services to resource-limited households, consistent with the pro-poor objectives of the national dairy development agenda.

The probit model results provide deeper insights into the micro-level drivers of income differentiation under FPT. The positive association of calf birth weight and average daily milk yield with net income highlights the dual pathway of genetic and management effects on household economics (Kabunga et al., 2017; Choudhury and Headey, 2018; DeLay, et al., 2020). Conversely, the negative influence of delayed reproductive maturity and prolonged service periods reveals the persistent bottlenecks in reproductive efficiency that undermine profitability when left unaddressed. The finding that cost per insemination exerted a significant negative impact on income further emphasizes the critical role of affordable and accessible breeding inputs. Together, these results corroborate the broader theoretical framing that livestock productivity and profitability are contingent not on isolated factors but on the integrated optimization of genetics, management, and input delivery (Da Costa Freitas and Barbosa, 2025; Vanvanhossou et al., 2021). The probit model also revealed that while some parameters such as calving interval showed modest positive associations, others like AI per conception did not significantly influence income in the matched sample, pointing to the heterogeneity of pathways through which reproductive performance translates into financial outcomes.

The causal inference results lend robust support to the conclusion that FPT participation substantially enhanced household welfare. The ATT estimates revealed a significant increase in net income per buffalo per annum for adopters, with results consistent across alternative estimation techniques, including IPWRA, PSM, and CEM. The convergence of results across these methods strengthens confidence in the robustness of treatment effects and rules out spurious correlations attributable to sample selection bias (Mwangi et al., 2021; Lu et al., 2021; Deme and Mangani, 2025). This finding aligns with global evidence that structured breeding programs, when coupled with field-level monitoring and data-driven selection, yield significant income gains for smallholder households (Rizzo et al., 2023). The estimated income differential of approximately ₹ 35,000 - ₹ 37,000 per buffalo per annum represents a substantial livelihood enhancement in the rural Indian context, where dairy often contributes a significant share of household cash flow and nutritional security. Such income increments are particularly consequential in regions with limited non-farm employment opportunities, underscoring the program’s role in inclusive rural development.

Beyond immediate economic gains, the study highlights broader implications for the sustainability of buffalo dairying. The genetic improvements achieved under FPT translate not only into higher productivity but also into enhanced resource-use efficiency. Shorter dry periods and reduced age at first calving imply lower feed and maintenance costs per unit of output, thereby improving feed conversion efficiency and reducing the environmental footprint of milk production (Boulton et al., 2017). These sustainability co-benefits are increasingly critical in the context of climate change and resource constraints, where the livestock sector is under pressure to deliver higher outputs with fewer inputs (Homann-Kee Tui et al., 2023). Furthermore, the premium commanded by genetically superior buffaloes in livestock markets 2024 to seek improved genetics, thereby expanding the diffusion of benefits beyond direct beneficiaries.

The policy relevance of these findings is significant. The demonstrable economic benefits of FPT justify continued and scaled-up investment in structured progeny testing as a foundation of the NPBI. However, the results also suggest the need for complementary interventions to address residual inefficiencies. For instance, while genetic improvements enhanced milk yields and reproductive efficiency, the persistence of extended service periods among some households indicates gaps in heat detection, veterinary access, and reproductive management practices (Bereket, 2024). Addressing these through targeted extension, training, and digital monitoring could further amplify program impacts. Moreover, the observation that adopters were disproportionately households with higher experience and marginally higher literacy points to the need for inclusive targeting strategies that ensure participation of younger and less-experienced farmers, who may otherwise remain excluded from the benefits of genetic improvement.

In broader perspective, the findings of this study contribute to the national discourse on dairy development. India’s position as the world’s largest milk producer is increasingly shaped by the performance of its buffalo population, particularly the Murrah breed. Enhancing genetic merit through structured FPT programmes offers a viable pathway to increase productivity while maintaining the adaptive strengths of indigenous breeds. Coupled with parallel investments in feed, health, and market infrastructure, such programmes can significantly elevate India’s dairy sector toward achieving nutritional security, export competitiveness, and rural income growth.

## 5 Conclusion

Advancing genetic improvement in buffalo populations remains central to strengthening dairy systems in South Asia and beyond, where buffaloes contribute substantially to rural livelihoods, nutritional security, and climate-resilient milk production. This study contributes to the emerging body of evidence that structured field progeny testing programs can serve as effective vehicles for translating genetic potential into tangible livelihood outcomes. By linking scientific breeding interventions with socio-economic realities at the household level, the findings emphasize that genetic progress is not merely a technical pursuit but a cornerstone of inclusive agricultural development. Globally, livestock research is increasingly challenged to deliver solutions that address productivity, profitability, and sustainability in tandem. The present work underscores that programs such as the National Program on Bovine Breeding in India hold relevance far beyond national borders, as they exemplify how public investments in genetic improvement, when combined with effective extension support, can drive systemic change. Importantly, these initiatives highlight the need for integrated approaches that simultaneously strengthen reproductive efficiency, reduce environmental footprints, and empower farming communities—particularly women, who are pivotal actors in buffalo husbandry.

A critical dimension in this context is the systematic evaluation of program impacts. Assessing whether investments in breeding interventions translate into measurable improvements in productivity, profitability, and social equity is vital for ensuring that public expenditure delivers genuine value. Such assessments provide not only evidence of effectiveness but also guidance for refining program design, targeting resources more efficiently, and sustaining long-term genetic gains. Viewed in this broader perspective, rigorous impact evaluation emerges as an indispensable instrument for policymakers and stakeholders, safeguarding accountability while reinforcing the role of buffalo genetic improvement as a catalyst for resilient and inclusive dairy development.

## 6 Limitations of the study

The findings of this study should be interpreted in light of certain limitations.

1. The analysis is geographically confined to Hisar district of Haryana, a region with a relatively advanced buffalo production system and well-developed veterinary infrastructure. Consequently, the estimated impacts may not be directly generalizable to regions with different agro-climatic conditions, herd structures, market access, or socio-economic contexts.
2. While robust quasi-experimental methods (IPWRA, PSM, and CEM) were employed to reduce observable selection bias, the analysis relies on a cross-sectional dataset and therefore cannot fully eliminate the influence of unobserved household-level factors such as farmer motivation, managerial ability, risk preferences, or informal knowledge networks. As with all observational studies, the estimated treatment effects are causal conditional on the assumption that no unobserved time-varying confounders simultaneously affect adoption and outcomes.
3. The estimated impacts represent average realized outcomes associated with current ownership of CIRB progenies, rather than cumulative genetic gains accruing over multiple generations since initial programme adoption. Due to herd turnover and the absence of reliable historical adoption timelines, intergenerational dose–response effects could not be explicitly modelled. Longitudinal herd-level data would be required to examine such dynamic genetic impacts.
4. The Field Progeny Testing programme operates as an integrated intervention combining superior germplasm dissemination with technical guidance and institutional support. The present study does not attempt to decompose the observed income gains into isolated effects of genetic improvement versus complementary services such as breeding support, health management, or advisory services. Accordingly, the results should be interpreted as the combined programmatic effect of the CIRB-FPT intervention under real-world implementation conditions, rather than as a pure genetic effect alone.

## Data Availability

The raw data supporting the conclusions of this article will be made available by the authors, without undue reservation.
