# Supplementary material for: Does adoption of superior Murrah buffalo germplasm pay off? evidence from a causal impact study in Haryana, India
Source: Front Genet. 2026 Jan 12;16:1713072. doi: 10.3389/fgene.2025.1713072 (PMC12832109; doi:10.3389/fgene.2025.1713072)
Supplement: Supplementary file 1 [file Table1.docx]

| 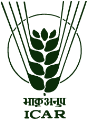 | ICAR-Central Institute for Research on Buffaloes, Hisar - 125001 | 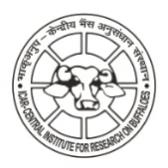 |
| --- | --- | --- |

| Interview schedule for dairy farmers (impact of dissemination of CIRB superior germplasm) | | | | | | | | | | | | | | | | | | | | | | | | | | | | |
| --- | --- | --- | --- | --- | --- | --- | --- | --- | --- | --- | --- | --- | --- | --- | --- | --- | --- | --- | --- | --- | --- | --- | --- | --- | --- | --- | --- | --- |
| 1. Name of the respondent: | | | | | | | |  | | | | | | | | | | | | **2. Mobile No.:** | | | | | |  | | |
| 3. Age in years: | |  | | | | | **4. Experience in dairy farming (years)** | | | | | | | | | | |  | | | | **5.Gender (M/F)** | | | | | |  |
| 6. Education (mark appropriate one) | | | | | | | | | | | Illiterate-0 | | | | Primary school (1-5^th^ Std)-1 | | | | | | | | | |  | | | |
| Higher primary  (6-8^th^ Std) -2 | | | High school  (8-10^th^ std)-3 | | | | | | | Pre university school (11&12) - 4 | | | | | | | Skill oriented course (Diploma/ITI)-5 | | | | | | Graduate & above-6 | | | | | |
| 7. Family size: | (Above 18): M …… F: …… | | | | | | | | | | | | | | | (Below 18): M: …… F: ……….. | | | | | | | | | | | | |
| 8. From which year started using CIRB bull semen (FPT prog.) | | | | | | | | | | | |  | | | | 9. How many progenies of CIRB do you have? | | | | | | | | | | |  | |
| 10. Main occupation: | | | | | Agriculture | | | | Dairying | | | | Business | | | Service | | | Agril. Labour | | | | | Others (labours, rural artisans) | | | | |
| Income (Rs. / annum): | | | | |  | | | |  | | | |  | | |  | | |  | | | | |  | | | | |
| 11. Subsidiary occupation: | | | | Agriculture | | | | | Dairying | | | | Business | | | Service | | | Agril. Labour | | | | | Others (labours, rural artisans) | | | | |
| Income (Rs. /annum): | | | |  | | | | |  | | | |  | | |  | | |  | | | | |  | | | | |
| 12. Where do you sell milk: | | | | | | Household/neighbours | | | | | Milk vendor | | | Halwies/sweet makers/tea stall | | | | | | | Dairy cooperatives | | | | others | | | |

**13. Land holding (acres)**

| Particulars | Irrigated | Rainfed | Total |
| --- | --- | --- | --- |
| Operational land holding |  |  |  |
| Area under fodder crops |  |  |  |

**14. Livestock inventory**

| Particular | Buffaloes  (in number) | Cows  (in number) | Total milk production (kg) | | Avg. value (Rs.) |
| --- | --- | --- | --- | --- | --- |
|  |  |  | **Buffaloes** | **Cows** |  |
| 1. In-milk |  |  |  |  |  |
| 2. Dry |  |  |  | |  |
| 3.Heifer  3.1. With pregnant  3.2. without pregnant |  |  |  | |  |
| 4. Calves |  |  |  | |  |
| 4.1 Male |  |  |  | |  |
| 4.2 Female |  |  |  | |  |

**15. Sale/purchase of buffaloes (last one year)**

| Items | Purchase | | Sale | |
| --- | --- | --- | --- | --- |
|  | Number | Average value | Number | Average value |
| 1. In-milk |  |  |  |  |
| 2. Dry |  |  |  |  |
| 3.Heifer  3.1. With pregnant  3.2. Without pregnant |  |  |  |  |
| 4. Calves  4.1 Male  4.2 Female |  |  |  |  |

**16. Details of breed-able female buffaloes**

| **S. No.** | **Parameters** | | | **Home born** | | | | | **Purchased** | | | | |
| --- | --- | --- | --- | --- | --- | --- | --- | --- | --- | --- | --- | --- | --- |
|  |  |  |  | **Buf-1** | **Buf-2** | | **Buf-3** | | **Buf-1** | | **Buf-2** | | **Buf-3** |
| 1 | Birth weight (kg) | | |  |  | |  | |  | |  | |  |
| 2 | Age at attainment of sexual maturity (years) | | |  |  | |  | |  | |  | |  |
| 3 | Dam’s milk yield (kg/lactation) | | |  |  | |  | |  | |  | |  |
| 4 | Age at first calving (months) | | |  |  | |  | |  | |  | |  |
| 5 | Present body weight (kg) | | |  |  | |  | |  | |  | |  |
| 6 | Number of calves born from buffaloes till date | | |  |  | |  | |  | |  | |  |
| 7 | Number of calves born alive from buffaloes till date | | |  |  | |  | |  | |  | |  |
| 8 | Lactation order (in number) | | |  |  | |  | |  | |  | |  |
| 9 | Last calving date (month & year) | | |  |  | |  | |  | |  | |  |
| 10 | First AI/Service after last calving (month) | | |  |  | |  | |  | |  | |  |
| 11 | After how many AI/service animal conceived (Numbers) | | |  |  | |  | |  | |  | |  |
| 12 | Name of the bull semen used in AI/service | | |  |  | |  | |  | |  | |  |
| 13 | Name of agency (CIRB/HLDB/Private/Don’t know) | | |  |  | |  | |  | |  | |  |
| 14 | Cost per AI/NS (includes semen straw+fees) | | |  |  | |  | |  | |  | |  |
| 15 | Dam’s milk yield of the bull semen used for AI/service | | |  |  | |  | |  | |  | |  |
| 16 | Average no. of service/AI per conception | | |  |  | |  | |  | |  | |  |
| 17 | Calving interval (months) | | |  |  | |  | |  | |  | |  |
| 18 | Duration of dry period (months) | | Previous lactation |  |  | |  | |  | |  | |  |
|  |  |  | Present lactation (exp.) |  |  | |  | |  | |  | |  |
| 19 | Average milk yield (kg.) | | Yesterday mor. |  |  | |  | |  | |  | |  |
|  |  |  | Yesterday eve. |  |  |  |  |  |  |  |  |  |  |
|  |  |  | Today mor. |  |  |  |  |  |  |  |  |  |  |
| 20 | Avg. Maximum milk yield/day (kg.) | | |  |  | |  | |  | |  | |  |
| 21 | Avg. Minimum milk yield/day (kg.) | | |  |  | |  | |  | |  | |  |
| 22 | Present value of the buffalo (in Rs.) | | |  |  | |  | |  | |  | |  |
| 23 | Lactation length (months) | | Previous lactation |  |  | |  | |  | |  | |  |
|  |  |  | Present lactation (exp.) |  |  | |  | |  | |  | |  |
| 24 | Lactation milk yield (kg) | | Previous lactation |  |  | |  | |  | |  | |  |
|  |  |  | Present lactation (exp.) |  |  | |  | |  | |  | |  |
| 25 | Standard lactation length milk yield (kg) | | Previous lactation |  |  | |  | |  | |  | |  |
|  |  |  | Present lactation (exp.) |  |  | |  | |  | |  | |  |
| **Production/reproduction related problems if any** | | | | | | | | | | | | | |
| 26 | Dystocia in last calving (Yes/No) | | |  |  |  | |  | |  | |  | |
|  | If yes how much money spent in treatment (Rs.) | | |  |  |  | |  | |  | |  | |
| 27 | Retention of placenta (Ys/No) (in last calving) | | |  |  |  | |  | |  | |  | |
|  | If yes how much money spent in treatment (Rs.) | | |  |  |  | |  | |  | |  | |
| 28 | Still birth in last calving (Yes/No) | | |  |  |  | |  | |  | |  | |
|  | If yes how much money spent in treatment (In Rs.) | | |  |  |  | |  | |  | |  | |
| 29 | a. Occurrence of mastitis (Yes/No) | Previous lactation | |  |  |  | |  | |  | |  | |
|  |  | Present lactation | |  |  |  | |  | |  | |  | |
|  | b. if yes how much money spent in treatment (Rs.) | Previous lactation | |  |  |  | |  | |  | |  | |
|  |  | Present lactation | |  |  |  | |  | |  | |  | |

**17. Income from farm**

| Type of Animal | Total milk production (Kg. /day.) | Consumption at farm/home (Kg/day) | Qty. Sold | Agency to Whom  Sold | | Sale price of milk (Rs. /kg.) |
| --- | --- | --- | --- | --- | --- | --- |
|  |  |  |  | **Qty (kg/day)** | **Name of Agency** |  |
| Buffalo |  |  |  |  |  |  |
| Cow |  |  |  |  |  |  |
| Total |  |  |  |  |  |  |

**18. Miscellaneous income**

| Particulars | No. of Qty per year | Rate (Rs/kg or unit) | Total amount |
| --- | --- | --- | --- |
| 1)Breeding services- Buffaloes |  |  |  |
| 2) Sale of dung (quintals) |  |  |  |
| 3) Sale of milk products-  a. Ghee (Buffalo/cow) |  |  |  |
| b. Curd |  |  |  |
| c. other products |  |  |  |
| 4) Any other (Rs. ) |  |  |  |

**19. Cost of feed and fodder (summer and winter season) (per farm)**

| Type of fodder/feed | Summer | | | Winter | | |
| --- | --- | --- | --- | --- | --- | --- |
|  | **Qty / day** | **Cost of production (Rs. / kg)** | **Purchase Rate** | **Qty / day** | **Cost of production (Rs. / kg)** | **Purchase Rate** |
| Green fodder |  |  |  |  |  |  |
| Dry fodder |  |  |  |  |  |  |
| Concentrate |  |  |  |  |  |  |
| Mineral mix |  |  |  |  |  |  |

**20. Inventory of the dairy farm**

| S. No. | Item name | Area/Number | Purchase/construction year | Expected life span | Present value | Repair cost/year |
| --- | --- | --- | --- | --- | --- | --- |
| 1 | Animal shed |  |  |  |  |  |
| 2 | Store for feed and fodder |  |  |  |  |  |
| 3 | Chaff cutter shed |  |  |  |  |  |
| 4 | Chaff cutter  a. Manual  b. mechanic |  |  |  |  |  |
| 5 | Buckets |  |  |  |  |  |
| 6 | Cart/trolley |  |  |  |  |  |
| 7 | Tube well |  |  |  |  |  |
| 8 | Milk cans |  |  |  |  |  |
| 9 | Air cooler |  |  |  |  |  |
| 10 | Milking machine |  |  |  |  |  |
| 11 | Iron chain |  |  |  |  |  |

**21. Other expenditure**

| S. No. | Particular | Cost per year |
| --- | --- | --- |
| 1 | Artificial insemination |  |
| 2 | Natural service |  |
| 3 | Animal insurance |  |
| 4 | Veterinary charges (medication and vetry. doctor fees) |  |
| 5 | Water |  |
| 6 | Electricity |  |
| 7 | Fuel |  |
| 8 | Ropes, gunny bags, brooms |  |
| 9 | Identification tags |  |

**22. Improved buffalo husbandry practices adopted by farmers**

| S. No. | Particulars | Regular | Sometimes | Seldom |
| --- | --- | --- | --- | --- |
| 1 | AI in buffaloes | Regular | Sometimes | Seldom |
| 2 | Pregnancy diagnosis | Regular | Sometimes | Seldom |
| 3 | Vaccination | Regular | Sometimes | Seldom |
| 4 | Balanced feeding | Fully adopted (Grain + Cake + Bran + salt + MM) | Partially adopted (Grain + Cake + Bran) | No adopted (Cake/Grain) |
| 5 | Green fodder | Round the year | Kharif+Rabi | Never |
| 6 | Mineral mixture | Fully adopted in all animals | Partially adopted (In milch animals) | Not adopted |
| 7 | Deworming | Regular | Sometimes | Seldom |
| 8 | Colostrum feeding | Regular | Sometimes | Seldom |
| 9 | Vermicomposting | Regular | Sometimes | Seldom |
| 10 | Silage | Round the year | Kharif+Rabi | Never |
